# Transcriptomic Profiling of Bean Aphid *Megoura crassicauda* upon Exposure to the Aphid-Obligate Entomopathogen *Conidiobolus obscurus* (Entomophthoromycotina) and Screening of CytCo-Binding Aphid Proteins through a Pull-Down Assay

**DOI:** 10.3390/insects15060388

**Published:** 2024-05-27

**Authors:** Jiaqin Zhu, Yaqi Fu, Lvhao Zhang, Tian Yang, Xiang Zhou

**Affiliations:** 1 Jixian Honors College, Zhejiang A&F University, Hangzhou 311300, China; 202124060117@stu.zafu.edu.cn; 2 National Joint Local Engineering Laboratory of Biopesticide High-Efficient Preparation, College of Forestry & Biotechnology, Zhejiang A&F University, Hangzhou 311300, China; 2021102032001@stu.zafu.edu.cn (Y.F.); 2020102081016@stu.zafu.edu.cn (L.Z.); 2021102032004@stu.zafu.edu.cn (T.Y.)

**Keywords:** His-tag pull down assay, LC-MS/MS, pore-forming toxin, calcium imbalance, insect immunity

## Abstract

**Simple Summary:**

In agriculture, aphids pose a significant pest threat, necessitating the deployment of entomopathogenic microorganisms for control. Fungi from the Entomophthorales order are particularly promising for biological control due to their efficacy and sustainability. This study employed transcriptomic analysis and a pull-down assay to investigate the interaction between *Megoura crassicauda* and *Conidiobolus obscurus*, uncovering how the fungus impacts aphids early in infection. The findings suggest that *C. obscurus* suppresses aphid immunity and hyperactivates the neuromotor system, hastening lethal effects. The insecticidal CytCo protein plays a vital role in this phase via putatively interacting with a calcium-transporting ATPase in *M. crassicauda*, potentially leading to neurological toxicity.

**Abstract:**

Prolonged periods of host-lethal infection by entomopathogenic fungi pose challenges to the development of biological control agents. The obligate entomopathogen *C. obscurus*, however, rapidly kills aphid hosts, warranting investigation. This study investigated the interaction between *C. obscurus* and a bean aphid *Megoura crassicauda* during the incubation period of infection, using transcriptome analysis to map host gene expression profiles. Results indicate *C. obscurus*-inoculated aphid activation of the wound healing immune responses, alongside suppression of the key molecules involved in Toll signaling, melanization, and metabolism. Furthermore, neuromotor system-related genes were upregulated, paralleling the intoxication observed in a nematode pest treated with *C. obscurus*-derived CytCo protein. To deepen interaction insights, a His-tag pull-down assay coupled with mass spectrometry analysis was conducted using CytCo as a bait to screen for potential aphid protein interactors. The proteins were identified based on the assembled transcriptome, and eleven transmembrane proteins were predicted to bind to CytCo. Notably, a protein of putatively calcium-transporting ATPase stood out with the highest confidence. This suggests that CytCo plays a vital role in *C. obscurus* killing aphid hosts, implicating calcium imbalance. In conclusion, *C. obscurus* effectively inhibits aphid immunity and exhibits neurotoxic potential, expediting the infection process. This finding facilitates our understanding of the complex host–pathogen interactions and opens new avenues for exploring biological pest management strategies in agroforestry.

## 1. Introduction

Naturally occurring diseases in insects are predominantly triggered by a broad spectrum of fungal pathogens, notably those from the Hypocreales and Entomophthorales orders [1,2]. Unlike bacterial counterparts that depend on host feeding habits, these fungi exhibit an extraordinary capacity to quickly penetrate the cuticular barrier and infiltrate the host hemocoel via air-dispersed infective conidia [3], making them highly efficacious in managing sap-sucking pests like aphids and mosquitoes [4,5]. Of note, the entomophthoralean fungi frequently induce epizootics among natural insect populations due to their unique dissemination patterns [1]. It has been demonstrated that certain Hypocreales fungi, such as *Metarhizium anisopliae* and *Beauveria bassiana*, can be employed as environmentally friendly microbial control agents [6,7]; however, realizing consistent success in practical applications remains challenging. One major obstacle is the inherent delay between fungal penetration and host mortality, limiting their immediate impact [4]. 

Molecular biology advances have illuminated numerous genes, metabolic pathways, and secondary metabolites integral to fungus–insect interactions [2,8], fueling optimism for the development of more cost-effective mycoinsecticides for field pest management [9]. Genetic improvement of fungal virulence and stress resistance has been pursued, with the successful integration of both native and synthetic genes into Hypocreales genomes [6]. Notably, genes encoding neurotoxic peptides, cuticle-degrading proteases, and chitinases significantly amplify the fungi’s rapid infection and lethality against insect pests [10,11].

Obligate pathogens, in contrast to non-obligate generalists like most Hypocreales, demonstrate a higher virulence and reduced periods of lethal infection [12], attributed to their efficiency in suppressing host innate immunity and rapid intrahemocoelic replication. Some entomophthoralean fungi, such as *Entomophaga aulicae* which infects caterpillars, inhibit the activities of fungal glucan and chitin synthase, enabling rapid protoplast growth within the host and evading immune detection [3]. Their specialized nature may shorten periods of lethal infection through tailored adaptation to specific hosts, optimizing resource utilization and immune evasion strategies [13], yet the genomic intricacies of these specialist pathogens remain underexplored [14,15,16]. 

Host immunity and genetic resistance significantly impact the outcome of fungal infections [17,18,19], with insects deploying cellular and humoral immune responses post-penetration [2,19]. Circulating hemocytes initially counterattack, engaging in phagocytosis, aggregation, nodulation, and encapsulation, while the fat body that loosely associated cells lining the integument of the hemocoel orchestrates immune response through the secretion of antimicrobial peptides. These peptides appear in the hemolymph of challenged insects 6–12 h after the challenge. Activation of the prophenoloxidase cascade represents another facet of the humoral response [20]. Novel RNA interference strategies have emerged to inhibit host immunity, boosting the efficacy of biocontrol for agroforestry pests [19,21]. 

*Conidiobolus obscurus*, a specialized entomophthoralean pathogen targeting aphids, induces over 50% mortality within 24 h at a dosage of 1000 conidia/mm^2^ [22]. Its virulence factor, CytCo, a cytolytic-like protein, correlates directly with fungal virulence [23] and is implicated in hemocyte disruption, expediting aphid mortality [24]. The present study aims to elucidate the responses of the bean aphids *Megoura crassicauda* during the incubation period of *C. obscurus* infection based on transcriptomic analysis. To identify CytCo-interacting host proteins, a pull-down assay and liquid chromatography coupled with mass spectrometry analysis (LC-MS/MS) based on the assembled transcriptome have been conducted. This endeavor promises insights into the intricate host–pathogen interaction, facilitating advancements in developing biological control agents based on entomophthoralean fungi. 

## 2. Materials and Methods

### 2.1. Fungus Origin and Preparation

The isolate *C. obscurus* ARSEF 7217 was obtained from the USDA-ARS Collection of Entomopathogenic Fungal Cultures (ARSEF; Ithaca, NY, USA), and maintained under long-term storage at −80 °C via cryopreservation [25]. For culturing, *C. obscurus* was grown on rich Sabouraud dextrose agar plus yeast extract (SDAY: composed of 40 g/L dextrose, 10 g/L peptone, 10 g/L yeast extract, and 15 g/L agar; Sangon Biotech, Shanghai, China) in Petri dishes for 4 days at 24 ± 1 °C under a 12:12 h light/dark (L:D) photoperiod. The mashed culture pieces were transferred to 50 mL Sabouraud dextrose broth plus yeast extract (SDBY) and shaken at 120 rev/min for 4 days at 24 ± 1 °C in a 150 mL flask. Following filtration dan double washing, the SDBY cultures were evenly spread on 90 mm Petri dishes. Residual water was absorbed with sterile paper and the plates were maintained for 12 h at 24 ± 1 °C to promote sporulation before being used for aphid inoculation via conidial shower [8].

### 2.2. Aphid Inoculation Procedure

Alate adult *M. carassicauda* were captured using yellow cloth and plant traps on a building rooftop within the campus and subsequently transferred onto leaf-inclusive dishes for maintenance at 24 ± 1 °C under a 12:12 h light/dark (L:D) photoperiod [26]. The dishes were simply embedded with fresh detached leaves on 1.5% agar, with foliar undersides exposed for aphid nourishment. 

Approximately 50 apterous adults of *M. crassicauda* were placed in each 90 mm leaf-inclusive dish for inoculation. Sporulating fungal plates were inverted above the aphid-containing dishes, to shower onto the aphids. A glass coverslip positioned beneath each setup facilitated the quantification of deposited conidia, aiming for a density of ca. 100 conidia/mm^2^. To ensure uniform distribution, the plates were rotated 90° every quarter of the time of exposure to the conidial shower [22]. 

### 2.3. Sampling of Aphids and RNA Extraction

Post-inoculation, aphids were reared at 24 ± 1 °C for 12 h under saturated humidity before sampling. Healthy aphids, consisting of roughly 100 apterous adults each, were collected directly from plants and snap-frozen in liquid nitrogen. Three samples of healthy and inoculated aphids were used as replicates for RNA extraction, respectively.

RNA extraction and quality assessments adhered to the previous protocol [8]. Briefly, the total RNAs of each sample were separately extracted using the RNAiso Plus kit (TaKaRa, Tokyo, Japan). Concentration was measured on a NanoDrop2000 (Thermo Fisher Scientific, New York, NY, USA), and integrity was checked on an Agilent 2100 Bio-Analyzer (Agilent Technologies, Santa Clara, CA, USA). RNA degradation and contamination were monitored on 1% agarose gels, and the qualified samples were sent to Biomarker Technologies Co., Ltd. (Beijing, China) for transcript sequencing.

### 2.4. Transcriptome Analysis

Aphid mRNA was enriched from each total RNA sample using oligo(dT) magnetic beads. Paired-end RNA-seq libraries of different treatments were prepared following Illumina’s library construction protocol, and the libraries were then sequenced on the Illumina Hiseq 4000 platform (BGI, Beijing, China). The raw data were deposited in the CNGB Sequence Archive (CNSA) of the China National GeneBank Database (CNGBdb, https://db.cngb.org) (accessed on 24 May 2024) under the accession number CNP0003732 [27]. De novo assembly pooled the six samples together and was performed using Trinity [28]. The *M. crassicauda* uniproteins were prepared to facilitate the identification of potential CytCo-binding proteins. The R package RSEM was used to calculate the fragments per kilobase of exon per million fragments mapped (FPKM) values [29]. Differentially expressed genes (DEGs) between libraries were filtered using the R package DEGseq [30]. The resulting *p*-values were adjusted using the Benjamini–Hochberg method (multiple-hypothesis tests) for controlling the false discovery rate (FDR). Transcripts with an adjusted *p*-value of below 0.01 and an absolute value of log2 fold change (FC) exceeding 1 were designated as differentially expressed [30].

To deepen the understanding of *C. obscurus* working on aphids, functional predictions of DEGs were performed through annotations, using several databases. Specifically, the genes were annotated using the Basic Local Alignment Search Tool (BLASTx) with an E-value threshold of 10^−5^. The public databases of Uni-Prot (https://www.ebi.ac.uk/uniprot) (accessed on 26 May 2023), Pfam (http://pfam.xfam.org) (accessed on 26 May 2023), Gene Ontology (GO, www.geneontology.org) (accessed on 24 May 2024), and Kyoto Encyclopedia of Genes and Genomes (KEGG; www.genome.jp/kegg/kegg2.html) (accessed on 24 May 2024) databases were used [31]. Protein–protein interaction (PPI) of DEGs was predicted by blasting the genome of a related species in the STRING database (http://stringdb.org/) (accessed on 6 May 2024) and then visualized in Cytoscape [32].

### 2.5. The Real-Time Quantitative PCR (qPCR) Assay

Quantitative PCR was employed to validate the transcript abundance of selected DEGs. The total RNA (1 µg) of each sample was reverse-transcribed to cDNA using the PrimeScript^TM^ RT reagent kit with gDNA Eraser (TaKaRa, Kyoto, Japan). Next, the qPCR analysis of the cDNA samples was performed using SYBR Green PCR (SYBR Premix Ex Taq^TM^ II, TaKaRa), while the paired primers were designed and have been listed in Table S1. The PCRs were performed on a Real-Time PCR Thermal Cycler (qTOWER 2.2, Analytik Jena, Jena, Germany), while the data were analyzed using the qPCRsoft v1.1 software (Analytik Jena, Jena, Germany). Moreover, expression levels in healthy and inoculated aphids were quantified across at least three biological replicates. The fold change was normalized to the internal reference gene encoding elongation factor 1-alpha (*ef-1α*, primers EF1-F/R listed in Table S1), using the 2^−ΔΔCt^ method [8].

### 2.6. Pull-Down Assay

The purified CytCo protein with a 6× His (histidine) tag at the N-terminal was prepared according to the method [24]. For total protein extraction, 2 g of healthy aphids were ground in 5 mL of 50-mM sodium phosphate buffer (pH 8.0), 150 mM sodium chloride, 1 mM β-mercaptoethanol, 2 mM magnesium chloride, 5% glycerol, and 1% polyvinyl pyrrolidone (*w*/*v*) supplemented with 1/2 protease cocktail inhibitor EDTA free (Calbiochem, Darmstadt, Germany), and 2 μL of benzonase nuclease (Sigma-Aldrich, Darmstadt, Germany). The protein extract was incubated on ice for 15 min, centrifuged at 4000× *g* for 5 min at 4 °C, followed by a second centrifugation at 12,000× *g* for 15 min at 4 °C. The supernatant was filtered through a 0.45 μm syringe filter to remove insoluble particles.

The potential interacting partners of CytCo were identified using Dynabeads™ His-Tag isolation and pulldown (Invitrogen #10103D, Carlsbad, CA, USA) following the manufacturer’s instructions. CytCo, mixed with the aphid protein extract, was gently agitated at 4 °C for 2 h. Post-incubation, the mixture was filtered and loaded onto an equilibrated cobalt spin column. Nonspecifically bound proteins were removed using 15 column volumes of wash buffer (50 mM phosphate buffer, pH 7.4, 150 mM NaCl, 10 mM imidazole). As a control for nonspecific binding proteins to the affinity matrix, His-tag (Roche Life Science Products, Basel, Switzerland) alone was incubated with protein extract and processed identically. CytCo and its prey proteins were then eluted with 100 μL of His-Elution Buffer.

### 2.7. Identification of the Potential CytCo-Binding Proteins by LC-MS/MS

Eluted samples were subjected to 10% SDS-PAGE and Silver stained. Target protein bands were excised from the gel, washed for 10 min by ddH_2_O twice, reduced in 50 mM NH_4_HCO_3_/acetonitrile (1:1), and destained for 20 min at 37 °C. Then, samples were alkylated with 50 mM iodoacetamide in dark conditions, and digested overnight using 25 µg/mL trypsin (Promega Sequencing Grade Modified Trypsin, Madison, WI, USA). The peptides produced by the enzymatic cleavage were loaded onto a silica column packed with C18 reverse-phase resin (particle size, 1.9 µm; pore size, 150 Å). After desalting, peptides were separated using an analytical C18 column (75 µm × 100 mm; particle size, 1.9 µm) with a linear gradient of 8–30% Mobile Phase B (95% acetonitrile containing 0.1% formic acid) at 300 nL/min for 20 min. Mass spectra were acquired in positive-ion mode with automated data-dependent MS/MS on the five most intense ions detected in preliminary MS scans using a TripleTOF 6600 (Applied Biosystem, Carlsbad, CA, USA). MS/MS raw files were used to query the Uniprot public protein database and the assembled *M. crassicauda* uniproteins in ProteinPilot™ software (SCIEX, Framingham, MA, USA, version 5.0) utilizing the Paragon algorithms with the parameter of trypsin as the digested enzyme and detected protein threshold with the parameters of Unused ProtScore ≥ 1.3 (≥95% peptide confidence) and a minimum of two unique peptides (≥95% confidence) for protein identification. Considering CytCo as a pore-forming toxin on the membrane, the DeepTMHMM tool (https://dtu.biolib.com/DeepTMHMM) (accessed on 24 May 2024) was employed to evaluate potential transmembrane structures among interactors. The binding site between CytCo and the most possible interactor was visualized using PyMOL (https://pymol.org) (accessed on 24 May 2024).

## 3. Results

### 3.1. Transcriptome Assembly and Annotation

For the assembly of the *M. crassicauda* transcriptome, Illumina HisSeq 4000 generated reads from both healthy and inoculated aphid samples were utilized. In total, this yielded 143,540,381 high-quality clean reads following de novo sequencing (Table S2). These reads were then assembled using the Trinity program, yielding 39,855 unigenes with lengths exceeding 300 base pairs, encompassing a total of 46,962,763 bases. 

Subsequently, all unigene sequences were annotated by searching the Nr NCBI protein database using BLASTX with an E-value threshold of 10^−5^. Out of the 39,855 unigenes from *M. crassicauda*, 16,417 sequences demonstrated matches to known proteins, predominantly exhibiting the highest homology with *Acyrthosiphon pisum* (5495 hits), followed by *Myzus persicae* (2777), *Aphis craccivora* (1826), and *Aphis gossypii* (282). To categorize the functional prediction of *M. crassicauda* proteins, GO and KOG terms were employed. In particular, 12,170 unigenes (74.1%) showed strong homology (E value < 1 × 10^−5^) and were enriched for GO terms (Figure S1). Additionally, 8,469 (51.6%) unigenes were functionally annotated to KOG terms, spanning 25 distinct clusters (Figure S2).

### 3.2. Functional Enrichment Analysis of Differentially Expressed Genes

**Figure 1:**
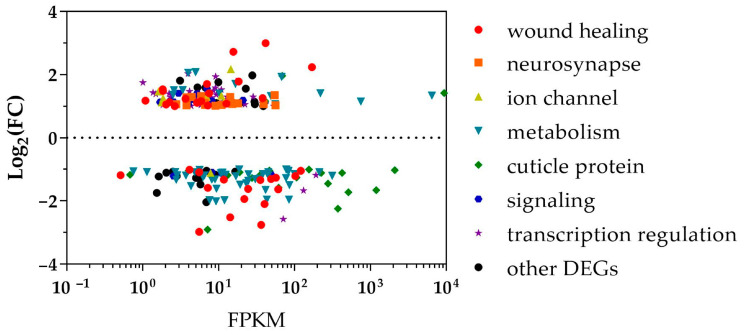
Differentially expressed genes of *Megoura carassicauda* in response to *Conidiobolus obscurus* infection during the early phase. A scatter plot shows the relationship between fragments per kilobase per million fragments (FPKM) and log fold change (log_2_FC) for each differentially expressed gene (DEG, FC ≥ 2) based on the sequencing of RNA extracted from healthy and inoculated *M. carassicauda*. Each symbol represents a single coding sequence; black circles indicate genes that are differentially expressed between the healthy and inoculated aphids at a false discovery rate of ≤0.01, and colored symbols indicate different functional groups of DEGs relative to the aphid–pathogen interaction.

To elucidate the impact of *C. obscurus* on *M. crassicauda* within the first 24 h post-inoculation, DEG analysis was conducted, revealing 463 DEGs (including 230 up-regulated and 233 down-regulated with a fold change ≥ 2) between inoculated and healthy aphids (Figures S3–S5). Upon consolidation of similar sequences, 351 (75.8%) DEGs were annotated, with 162 (35.0%) DEGs matching entries in the Uniprot database, 254 (55.1%) associated with GO terms, and 223 (48.2%) mapped to KEGG pathways. Unregulated DEGs were putatively implicated in processes such as wound healing (Table 1), signaling transduction (Table 2), and transcription regulation (Table S3). Downregulated DEGs were related to cuticle protein and metabolism (Figure 1). The RT-qPCR results validation largely concurred with the transcriptome findings in terms of expression trends, albeit with variations in the magnitude of relative expression levels (Figure S6).

**Figure 2:**
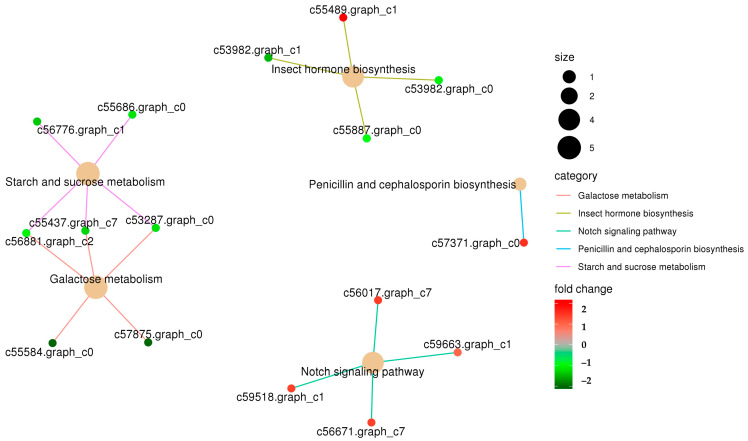
The interactive network of KEGG functional enrichment in the subcategory of the DEGs. Red: upregulated (FC ≥ 2), Green: downregulated. Size of dots related to the gene number.

**Figure 3:**
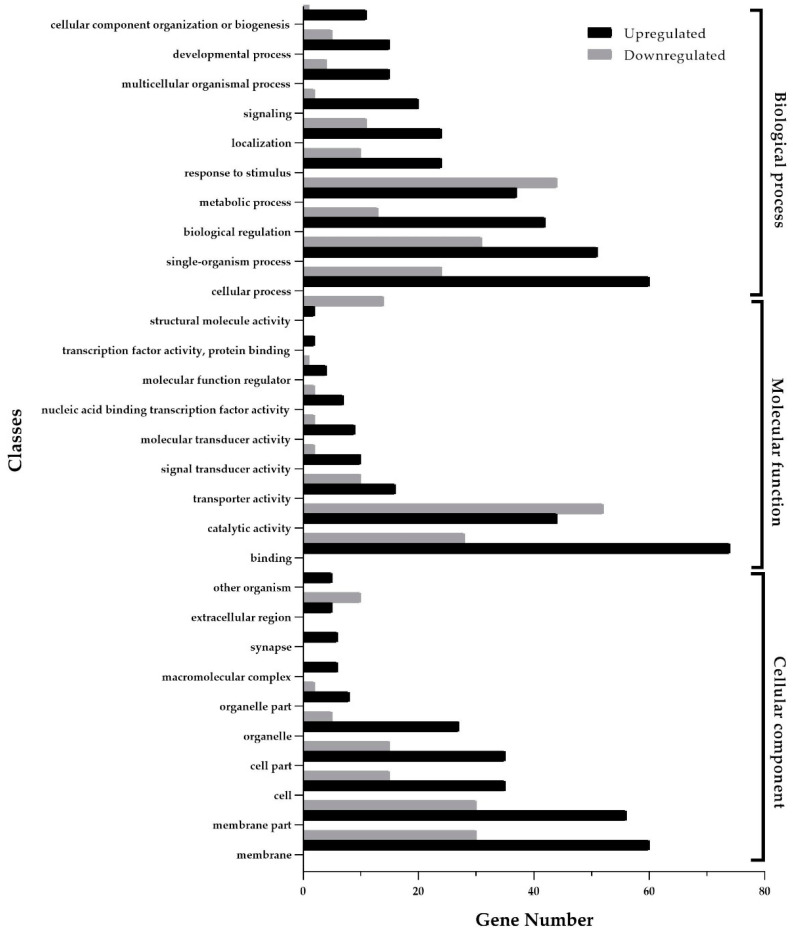
Differentially expressed genes between the healthy and *Conidiobolus obscurus*-inoculated aphids associated with Gene Ontology (GO) terms. The ordinate is GO classification grouped into three hierarchically stretched GO terms; the left abscissa represents the numbers of DEGs in GO classification. The black columns represent the numbers of upregulated DEGs and the gray columns stand for the numbers of downregulated DEGs.

The KEGG enrichment analysis highlighted the significant clustering of DEGs in several metabolic and signaling pathways, specifically starch and sucrose metabolism, galactose metabolism, insect hormone biosynthesis, and notch signaling pathways (Figure 2). As illustrated in Figure 3, the 254 GO-annotated DEGs were categorized into 57 secondary-level classifications across the three primary ontologies: molecular function (comprising 16 classes), cellular component (19 classes), and biological process (21 classes). The largest class of DEGs was binding (including 74 DEGs upregulated and 28 downregulated, Table S4), followed by the classes of catalytic activity (44 upregulated and 52 downregulated) in molecular function and membrane (60 upregulated and 30 downregulated) in the cellular component.

### 3.3. Identification of CytCo-Binding Proteins

**Figure 4:**
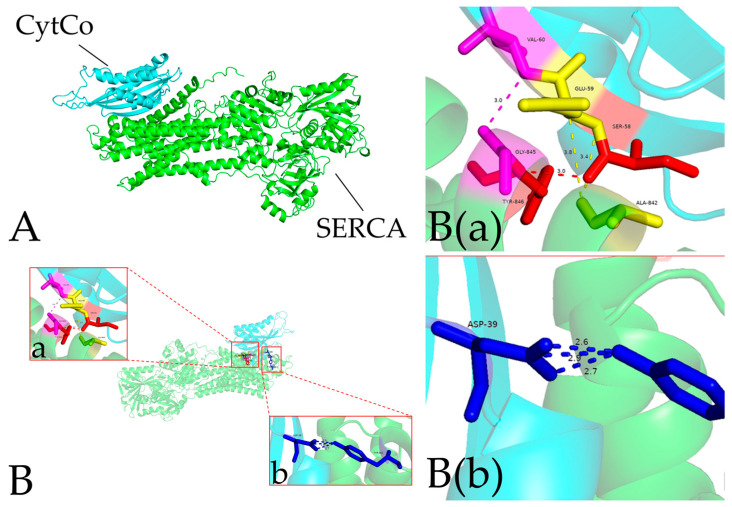
The computational model of the interaction between the neighboring subunits of CytCo and SERCA. (**A**): 3D structures of CytCo (in blue color) and sarcoplasmic/endoplasmic reticulum calcium-transporting ATPase (SERCA, in green color). The confidence score of protein docking was 0.93. (**B**): Hydrogen bonds between CytCo and SERCA molecules are shown as colored dashed lines, including four potential amino acid binding sites: the 58th Serine of CytCo with the 846th Tyrosine of SERCA, the 39th Aspartate of CytCo with the 861st Tyrosine of SERCA, the 59th Glutamate of CytCo with the 842nd Alanine of SERCA, and the 60th Valine of CytCo with the 845th Glycine of SERCA. B(a) b shows the amino acid hydrogen bonding information of region a in B. B(b) shows the amino acid hydrogen bonding information of region b in B.

To further investigate the role of CytCo during the initial stage of infection, a pull-down assay was employed to screen the CytCo-binding aphid proteins. Following incubation of purified CytCo with the protein extract, unbound aphid proteins were removed. The sample was exhaustively washed to remove all unbound proteins, and each eluted fraction was analyzed by SDS-PAGE until no protein was detectable in the eluent. Proteins that remained bound to the CytCo-coupled matrix were then dissociated and resolved via SDS-PAGE (Figure S7). In the LC-MS/MS analysis, a His-tag-only control was included to eliminate the false interactors. From this analysis, 80 candidate CytCo-binding proteins from *M. carassicauda* were identified, such as NADH dehydrogenase, fatty acyl-CoA reductase, ATP-dependent helicase, ecdysone, and venom protease (Table S5). Using the DeepTMHMM tool (Figure S8), 11 transmembrane proteins were predicted to potentially interact with CytCo, including calcium-transporting ATPase (SERCA), member of the transmembrane 9 family, synaptotagmin, ubiquitin-like modifier-activating enzyme (Table 3). Based on the criteria of ProtScore, sequence coverage, and peptide count, SERCA emerged as the most possible interactor of CytCo. To further elucidate the interaction predictions between CytCo and SERCA, three-dimensional (3D) models of both proteins were conducted and subjected to docking analysis, revealing four specific loci where PPI occurs (Figure 4).

## 4. Discussion

The present study reveals the intricate interaction between the obligate aphid pathogen, *C. obscurus*, and its host, *M. carassicauda*, during the incubation period of infection. While the aphid triggers immune responses, *C. obscurus* counteracts by suppressing host defenses and altering physiological functions. Invasion through the cuticle triggers an upregulated expression of immunoglobulin-like molecules, fibronectin, and mucins in the infected aphids, potentially signifying wound-healing processes. The upregulation of transcription factors, such as zinc finger proteins, Toll-like receptors, JNK interactors, Notch pathway components, and other signaling molecules, suggest intricate regulation of immune gene expression in the host. Given the downregulation observed in most cuticle protein-encoding genes in this study, it is conceivable that the upregulated ecdysone-induced transcription factors play a pivotal role in mediating aphid immunity, consistent with ecdysone’s role in *Drosophila* immunity [33].

*C. obscurus* displays a remarkable ability to suppress the host’s innate immune system. For example, we observed a downregulation of unigenes encoding phenoloxidase and phenoloxidase-activating enzymes, putatively relative to melanization [34]. Notably, pea aphids miss genes critical for the recognition, signaling, and elimination of microbial invaders in their genomes [34]. It implies that the frail aphid immune system may be more susceptible to disruption when key genes are suppressed. In our study, two unigenes encoding modular serine protease (ModSP) were downregulated. These enzymes function in activating the Toll pathway through the recognition of the invading fungi [35]. This suggests that *C. obscurus* inhibits the host Toll signaling pathway and the innate immune responses by downregulating key functional genes, thereby enabling its successful colonization. This not only highlights potential targets for enhancing the efficacy of entomopathogenic fungi in aphid control but also underscores the sophisticated mechanisms by which *C. obscurus* undermines host defenses.

Entomopathogenic fungi, such as their plant pathogen counterparts, deploy effector-like proteins and metabolites to evade or manipulate host immunity [2]. Hypocreales fungi, for instance, produce a diverse array of immunosuppressive metabolites designed to interfere with insect immune responses [9,19]. *B. bassiana* produces cytotoxic compounds such as beauvericin, beauveriolides, and benzoquinone oosporein to expedite fungal colonization [2]. Similarly, *M. anisopliae* secretes cyclopeptide destruxins that deactivate prophenoloxidases and suppress antimicrobial peptide genes by targeting immunophilins [19]. However, detailed information regarding how Entomophthorales overcome insect host immunity remains limited. Protein coronatin-2 and metabolites harman and norharman, produced by *Conidiobolus coronatus*, are known to cause cell death of *G. mellonella* hemocytes, thus facilitating successful fungal infection and host killing [36,37]. Additionally, *C. obscurus* CytCo has been proven to deliberately eliminate the insect hemocytes via the induction of apoptosis and necroptosis [24].

In this study, the changes in host gene expression during the early infection phase are comparable to the impacts of purified CytCo on gene expression in the hemocytes of *Galleria mellonella* and *Bursaphelenchus xylophilus* [24,38]. For example, suppressing the katabolic metabolism activities maximizes the host resources available for the pathogen [24]. The upregulation of many neurosynaptic and ion-channel-related genes, which are involved in the neuro-motor system, is probably attributed to the intoxication induced by CytCo. This aligns with the CytCo effects on *B. xylophilus* [38] and concurs with the observed paralysis in aphids inoculated with high conidial concentration of *C. obscurus*. It implies that *C. obscurus* exerts neurotoxic effects on its hosts via CytCo.

The pull-down assay revealed various potential CytCo interactors in aphids, notably the calcium-transporting ATPase on the sarcoplasmic/endoplasmic reticulum emerging as the most credible candidate. SERCA regulates voltage-gated calcium channels and calcium-activated potassium channels that together govern muscle excitability. Impaired SERCA function can lead to pathological conditions [39]. The inhibition of SERCA negatively affects calcium sequestration and reduces the magnitude of excitatory junctional currents at motor nerve terminals. *Drosophila* SERCA mutants present conditional paralysis due to prolonged muscle contraction driven by neural activity [40]. It suggests that CytCo affects the insect neuro-motor system via disrupting ion homeostasis, necessitating further work to confirm.

In conclusion, *C. obscurus* significantly influences the immune and neuromotor systems of *M. carassicauda* during the incubation phase, with CytCo playing a central interactive role. This deepens our comprehension of the host–pathogen interaction and development of aphid control measures.

## Figures and Tables

**Table 1:** The *Megoura carassicauda* immunity-related DEGs with FPKM values in inoculated aphids vs. healthy aphids ^a^.

Unigene ID	Annotation	FPKM		Log_2_(FC) ^b^	GO/KO ID
		Healthy	Inoculated		
**Immunoglobulin**					
c57868	Immunoglobulin I-set domain	1.81	6.99	1.70	K23915
c56461	Immunoglobulin V-set domain	0.39	1.84	1.48	K06772
c58593	Immunoglobulin V-set domain	1.33	3.66	1.23	GO:0016021
c56079	Immunoglobulin I-set domain	2.33	5.85	1.17	GO:0016021
c54209	Immunoglobulin V-set domain	0.84	2.25	1.13	K22655
c56508	Immunoglobulin I-set domain	1.06	2.64	1.01	GO:0016021
**Peroxidase or ROS-related**					
c55353 *	Chorion peroxidase	7.95	4.12	−1.01	K19511
c59130	Animal haem peroxidase	245.04	120.77	−1.04	K19511
c59021	Melanization protease	125.49	56.73	−1.27	GO:0004252
c55437	Phenoloxidase-activating enzyme	120.31	35.41	−1.34	GO:0004252
c58805	Glutamate transporter polyphemus	25.40	7.20	−1.58	K14209
**Wound healing**					
c54775	Fibronectin type III	5.48	18.32	1.78	K17591
c51444	Mucin 2-like protein	0.23	1.08	1.17	/
c54290	Fibronectin type III	2.55	6.57	1.11	GO:0005515
c57232	Ig-like and fibronectin type-III	5.32	12.58	1.09	K06774
c52952	Fibronectin type III	1.68	0.64	−1.18	GO:0002376
**Toll pathway**					
c57284	NAD(+) hydrolase sarm1	2.31	7.01	1.56	GO:0034128
c53917	Toll-like receptor	0.88	2.09	1.05	K10170
c54414	modular serine protease ModSP	84.80	34.60	−1.29	K20674
c57804	modular serine protease ModSP	34.66	7.57	−1.97	K20674
c58774	Serpin	76.40	21.80	−1.93	K13963
**Ecdysone-mediated**					
c55917	Krueppel 1-like	3.87	16.90	1.78	K09228
c56481	Ecdysone-induced protein	1.65	5.14	1.27	K08701
c55601	PR domain zinc finger protein 1 isoform X1	9.68	22.20	1.06	GO:0035075

^a^ Inoculated refers to the library constructed by transcripts of *M. carassicauda* 24 h after conidial inoculation. Healthy refers to the library constructed from the control treatment. The transcript level is expressed in fragments per kilobase per million fragments (FPKM) values. * Indicates potential interactors of the CytCo-binding aphid proteins screened by pull-down assay and LC-MS/MS analysis. ^b^ FC means fold change of differentially expressed genes (DEGs) between the two libraries.

**Table 2:** The *Megoura carassicauda* signaling transduction-related DEGs of with FPKM values in inoculated aphids vs. healthy aphids ^a^.

Unigene ID	Annotation	FPKM		Log_2_(FC) ^b^	GO/KO ID
		Healthy	Inoculated		
**Signaling**					
c56897	inositol 1,4,5-trisphosphate receptor	0.76	2.95	1.41	K04958
c56671	Protein serrate	1.75	5.11	1.39	GO:0007219
c58773	beta-1 adrenergic receptor	1.70	5.26	1.39	GO:0004930
c58924	receptor-type tyrosine-protein phosphatase	1.89	4.44	1.21	K06776
c55654	Rho GTPase-activating	8.45	20.90	1.19	K20655
c54354	ephrin-B1	2.89	8.17	1.17	K05463
c57822	G-protein coupled receptor	1.05	2.28	1.16	GO:0004930
c57839	FMRFamide receptor	0.64	1.74	1.14	GO:0008528
c54122	WNT1-inducible-signaling pathway	0.73	2.55	1.14	K22471
c56120	G-protein coupled receptor	0.57	1.68	1.13	K08469
c56130	regulator of G-protein signaling	17.00	40.40	1.11	GO:0001965
c57791	dual 3′,5′-cyclic-AMP and -GMP phosphodiesterase	4.82	11.60	1.08	GO:0004114
c56800	JNK-interacting	4.89	10.20	1.06	K20317
c54894	arrestin homolog	1.55	3.98	1.05	K04439
**Neuromotor system**					
c56627	Neuropilin and tolloid-like	5.08	14.20	1.29	K19404
c57981	unc-80 homolog	1.16	3.76	1.28	K24015
c54290	Neural cell adhesion	2.55	6.57	1.11	K06491
c57640 *	synaptotagmin 1 isoform X1	7.61	17.90	1.09	GO:0007269
c56243	Acetylcholine receptor subunit beta-like	18.70	39.30	1.07	K05312
c55778	neuronal acetylcholine receptor subunit α-7	8.41	15.10	1.07	GO:0022848
c59372	neurobeachin isoform X3	7.78	10.90	1.04	K24183
c58381	locomotion-related protein Hikaru genki	3.02	6.64	1.04	K17495
c59504	regulating synaptic membrane exocytosis	25.30	55.90	1.03	K15297
c55640	serine/threonine-protein kinase Nek8	1.64	3.77	1.03	K20877
c57984	myelin regulatory factor-like	4.91	9.05	1.02	/
**Ion channel**					
c58440	Voltage-gated chloride channel	2.11	14.4	2.17	GO:0005247
c58546	voltage-dependent calcium channel	0.35	1.61	1.47	K04863
c58915	Cyclic nucleotide-gated cation channel α-3	4.15	10.80	1.33	GO:0005249
c55895	Potassium channel domain	2.50	6.95	1.30	/
c57081	Small conductance calcium-activated potassium channel	2.32	5.82	1.30	GO:0016286
c56821	potassium voltage-gated channel	0.53	1.85	1.25	GO:0008076
c56460	Potassium channel	4.94	11.80	1.16	GO:0005267
c58522	potassium voltage-gated channel	0.66	1.77	1.12	K04905
c52823	glutamate-gated chloride channel-like	17.10	7.82	−1.11	GO:0005230

^a^ Inoculated refers to the library constructed by transcripts of *M. carassicauda* 24 h after conidial inoculation. Healthy refers to the library constructed from the control treatment. The transcript level is expressed in fragments per kilobase per million fragments (FPKM) values. * indicates potential interactors of the CytCo-binding aphid proteins screened by pull-down assay and LC-MS/MS analysis. ^b^ FC means fold change of differentially expressed genes (DEGs) between the two libraries.

**Table 3:** The potential interactors of *Megoura carassicauda* proteins binding to CytCo ^a^.

Unigene ID	Annotation	Accession No.	Unused ProtScore	Coverage (%)	Peptides	TMRs
c55126	calcium-transporting ATPase sarcoplasmic/endoplasmic reticulum type isoform X2	gi|1028717492	37.86	24.9	20	8
c55766	AMP deaminase 2 isoform X2	gi|1028703692	12.87	9.4	7	2
c57640	synaptotagmin 1 isoform X1	gi|641664799	9.68	14.4	5	1
c58629	ATP-binding cassette sub-family F member 1	gi|193676397	6.00	3	3	3
c52852	dihydroorotate dehydrogenase (quinone), mitochondrial	gi|641676995	5.23	8.3	3	4
c55131	probable hydroxyacid-oxoacid transhydrogenase, mitochondrial	gi|641678166	4.21	4.1	2	10
c53223	innexin inx7	gi|328723643	4.08	6.7	2	5
c59449	transmembrane 9 superfamily member 2	gi|1028716222	4.00	3.8	2	9
c52282	ubiquitin-like modifier-activating enzyme 5	gi|193707023	4.00	3.4	3	2
c57885	Mo25 uncharacterized	gi|328720151	3.64	5.8	2	6
c54081	tumor suppressor candidate 3	gi|641672251	1.49	5.8	2	6

^a^ Threshold with the parameters of Unused ProtScore ≥ 1.3 (corresponding to peptide confidence ≥ 95%) and at least two unique peptides (confidence ≥ 95%).

## Data Availability

Illumina sequence data have been submitted to CNGBdb (https://db.cngb.org/) (accessed on 24 May 2024) under the accession number CNP0003732. All data generated or analyzed during this study are included in this published article and Supplementary File.

## Supplementary Materials

The following supporting information can be downloaded at: https://www.mdpi.com/article/10.3390/insects15060388/s1, Table S1. The designed primers for qPCR. Table S2. The numbers of mapped reads from the six samples of Megoura crassicauda. Table S3. The Megoura carassicauda transcription regulation-related DEGs with FPKM values in inoculated aphids vs. healthy aphids. Table S4. The Megoura carassicauda binding-related DEGs in inoculated aphids vs. healthy aphids. Table S5. The potential aphid proteins binding to CytCo by pull-down assay and LC-MS/MS. Figure S1. GO classification of DEG. Figure S2. KOG Function Classification of Consensus Sequence. Figure S3. PCA analysis of two groups of samples. Figure S4. Expression levels of the differentially expressed genes among the six samples of Megoura crassicauda. Figure S5. Heat map of the transcription patterns of the differentially expressed genes among the six samples of Megoura crassicauda. Figure S6. RT-qPCR result of Megoura crassicauda DEGs’ relative quantification. Figure S7. SDS-PAGE of aphid proteins and CytCo-binding interactors. Figure S8. The transmembrane topology prediction of the potential CytCo-binding aphid proteins by DeepTMHMM.
