# Supplementary material for: Transcriptomic Profiling of Bean Aphid Megoura crassicauda upon Exposure to the Aphid-Obligate Entomopathogen Conidiobolus obscurus (Entomophthoromycotina) and Screening of CytCo-Binding Aphid Proteins through a Pull-Down Assay
Source: Insects. 2024 May 27;15(6):388. doi: 10.3390/insects15060388 (PMC11203964; doi:10.3390/insects15060388)
Supplement: Supplementary file 1 [file insects-15-00388-s001.zip › insects-3026599-supplementary.pdf]

Table S1 the designed primers for qPCR

Internal ID/gene	Primer Sequence	
	Forward	Reverse
c54162	CCAACCAATTACCTCAATC	TGCCTCCGATGTATTTAG
c56956	TTATTGTGTATAGTGCCATC	TAAGTCGGTATTGTGCTA
c59130	TAAGTAGGTATTAGCCATA	GCATATTGATTATCGTTAC
EF1	CGATACCTCCCTCCGCCCAG	ACAGGCGGAACGACCACAAC

Table S2. The numbers of mapped reads from the six samples of *Megoura crassicauda*

BMK-ID	Clean Reads	Mapped Reads	Mapped ratio
Healthy1	21351177	18400729	86.18%
Healthy 2	24791142	21308039	85.95%
Healthy 3	20542211	17100964	83.25%
Inoculation1	21121469	17390057	82.33%
Inoculation 2	29794858	25434002	85.36%
Inoculation 3	25939524	20289347	78.22%

Tables S3 The *Megoura carassicauda* transcription regulation-related DEGs of with FPKM values in inoculated aphids vs. healthy aphids [†]

Internal ID	Annotation	FPKM		$Log_2(FC)$[‡]	GO/KO ID
		Healthy	inoculated		
**Transcriptional regulator**					
c58259	ATP-dependent helicase brm	1.37	9.11	1.93	GO:0006355
c55697	Helicase domino	2.35	8.48	1.49	K11320
c57530	CCAAT/enhancer-binding	3.17	10.8	1.52	GO:0003700
C56017	histone acetyltransferase	0.19	1.36	1.43	K04498
c58145	Transcriptional regulator ATRX homolog	0.53	2.22	1.39	K10876
c59518	Aspartic peptidase domain	0.12	1.48	1.38	GO:0004190
c59663	RNase H-like domain	24.5	53.6	1.00	GO:0003676
c56504	myocardin-related transcription factor A	1.37	9.11	1.93	GO:0006357
**Zinc finger**					
c55891	Zinc finger, C2HC type	0.76	3.94	2.03	K23193
c56589	zinc finger CCCH	0.09	1	1.75	GO:0008168
c54162	Zinc-finger double domain	1.9	9.06	1.58	K09228
c58416	nuclear hormone receptor HR3	0.88	4.12	1.47	K14033
c56534	Zinc finger protein basonuclin-2	1.8	6.55	1.36	K24146
c58516	GATA zinc finger	3.01	9.31	1.36	/
c54361	zinc finger homeobox	2.32	6.69	1.32	K09378
c54162	C2H2-type zinc finger	8.06	28.4	1.30	K09228
c53080	zinc finger	0.48	2.35	1.28	K09219

[†] Inoculated refers to the library constructed by transcripts of *M. carassicauda* 12 h after conidial inoculation. Healthy refers to the library constructed from control treatment. Transcript level is expressed in fragments per kilobase per million fragments (FPKM) values.

[‡] FC means fold change of differentially expressed genes (DEGs) between the two libraries.

Table S4. The *Megoura carassicauda* binding-related DEGs in inoculated aphids vs. healthy aphids [†]

Internal ID	GO term	$Log_2(FC)$[‡]
c52908	protein binding (GO:0005515)	2.99
c55891	zinc ion binding (GO:0008270)	2.03
c58259	ATP binding (GO:0005524)	1.93
c55338	GTP binding (GO:0005525	1.93
c57757	actin filament binding (GO:0051015)	1.81
c55917	nucleic acid binding (GO:0003676)	1.78
c54775	protein binding (GO:0005515)	1.78
c57868	calcium ion binding	1.70
c47252	ATP binding (GO:0005524)	1.60
c47121	zinc ion binding (GO:0008270)	1.59
c54162	nucleic acid binding (GO:0003676)	1.58
c57530	DNA-binding transcription factor activity (GO:0003700)	1.52
c56118	metal ion binding (GO:0046872)	1.51
c55697	ATP binding (GO:0005524)	1.49
c58416	zinc ion binding (GO:0008270); sequence-specific DNA binding (GO:0043565)	1.47
c56017	zinc ion binding (GO:0008270)	1.43
c58281	protein binding (GO:0005515)	1.42
c57878	ATP binding (GO:0005524)	1.41
c56897	inositol 1,4,5 trisphosphate binding (GO:0070679)	1.41
c53754	nucleic acid binding (GO:0003676)	1.41
c56236	metal ion binding (GO:0046872)	1.40
c58145	ATP binding (GO:0005524)	1.39
c56671	calcium ion binding (GO:0005509)	1.39
c58773	RNA binding (GO:0003723)	1.39
c54890	flavin adenine dinucleotide binding (GO:0050660)	1.32
c54361	sequence-specific DNA binding (GO:0043565)	1.32
c55310	nucleic acid binding (GO:0003676);chaperone binding (GO:0051087)	1.31
c54162	nucleic acid binding (GO:0003676)	1.30
c57081	calmodulin binding (GO:0005516)	1.30
c56504	protein binding (GO:0005515)	1.30
c57021	DNA binding (GO:0003677)	1.30
c55889	GTP binding (GO:0005525)	1.29
c54689	nucleic acid binding (GO:0003676)	1.28
c56517	zinc ion binding (GO:0008270)	1.28
c56481	DNA-binding transcription factor activity (GO:0003700); zinc ion binding (GO:0008270); sequence-specific DNA binding (GO:0043565)	1.27
c53127	odorant binding (GO:0005549)	1.22
c55654	protein binding (GO:0005515)	1.19
c51444	chitin binding (GO:0008061)	1.18
c55712	protein binding (GO:0005515)	1.17
c55307	transcription regulatory region sequence-specific DNA binding (GO:0000976); regulatory region nucleic acid binding (GO:0001067); cis-regulatory region sequence-specific DNA binding (GO:0001158); nucleic acid binding (GO:0003676);DNA binding (GO:0003677)	1.17
c57530	DNA-binding transcription factor activity (GO:0003700)	1.16
c59686	nucleic acid binding (GO:0003676)	1.15

c54122	insulin-like growth factor binding (GO:0005520)	1.14
c59121	zinc ion binding (GO:0008270); Hsp70 protein binding (GO:0030544)	1.14
c55819	protein binding (GO:0005515)	1.14
c57336	sequence-specific DNA binding (GO:0043565)	1.13
c55505	ATP binding (GO:0005524)	1.13
c56206	actin binding (GO:0003779); ATP binding (GO:0005524)	1.12
c56130	G-protein alpha-subunit binding (GO:0001965)	1.11
c54290	protein binding (GO:0005515)	1.11
c59406	nucleic acid binding (GO:0003676)	1.11
c56958	ATP binding (GO:0005524)	1.10
c56584	DNA binding (GO:0003677)	1.10
c57884	protein binding (GO:0005515)	1.09
c57640	calcium-dependent phospholipid binding (GO:0005544)	1.09
c56915	DNA binding (GO:0003677)	1.09
c57791	metal ion binding (GO:0046872)	1.08
c56800	binding (GO:0005488);protein binding (GO:0005515)	1.06
c59312	ATP binding (GO:0005524)	1.06
c55601	DNA-binding transcription factor activity, RNA polymerase II-specific (GO:0000981); DNA-binding transcription factor activity, RNA polymerase II-specific (GO:0000982); DNA-binding transcription repressor activity, RNA polymerase II-specific (GO:0001078); DNA-binding transcription repressor activity, RNA polymerase II-specific (GO:0001227)	1.06
c59553	nucleic acid binding (GO:0003676)	1.06
c54847	nucleic acid binding (GO:0003676)	1.06
c51695	nucleic acid binding (GO:0003676); DNA binding (GO:0003677); double-stranded DNA binding (GO:0003690); heterocyclic compound binding (GO:1901363)	1.05
c58350	DNA binding (GO:0003677); zinc ion binding (GO:0008270)	1.04
c55592	regulatory region nucleic acid binding (GO:0001067)	1.04
c59504	Rab GTPase binding (GO:0017137); metal ion binding (GO:0046872)	1.04
c56414	calcium ion binding (GO:0005509)	1.03
c54398	DNA binding (GO:0003677)	1.03
c55640	ATP binding (GO:0005524)	1.03
c57984	DNA binding (GO:0003677)	1.02
c57847	nucleic acid binding (GO:0003676)	1.01
c55122	SMAD binding (GO:0046332)	1.01
c59663	nucleic acid binding (GO:0003676)	1.00
c59719	actin binding (GO:0003779); calcium ion binding (GO:0005509); microtubule binding (GO:0008017)	1.00
c59697	DNA binding (GO:0003677)	-1.00
c55353	heme binding (GO:0020037)	-1.01
c57707	protein binding (GO:0005515)	-1.04
c59130	heme binding (GO:0020037)	-1.04
c47259	NADP binding (GO:0050661); NAD binding (GO:0051287)	-1.07
c58653	metal ion binding (GO:0046872)	-1.09
c55314	protein binding (GO:0005515)	-1.09
c54551	metal ion binding (GO:0046872)	-1.13
c57583	DNA-binding transcription factor activity (GO:0003700)	-1.14
c58544	iron ion binding (GO:0005506)	-1.15
c55686	cation binding (GO:0043169)	-1.17

c52952	actin binding (GO:0003779); protein binding (GO:0005515)	-1.18
c57694	chitin binding (GO:0008061)	-1.18
c47211	lipid binding (GO:0008289)	-1.19
c53969	calcium ion binding (GO:0005509)	-1.20
c49312	DNA-binding transcription factor activity (GO:0003700)	-1.25
c56501	DNA binding (GO:0003677)	-1.27
c58051	flavin adenine dinucleotide binding (GO:0050660)	-1.31
c58735	heme binding (GO:0020037);;	-1.32
c50559	zinc ion binding (GO:0008270)	-1.32
c56512	lipid binding (GO:0008289)	-1.33
c47099	lipid binding (GO:0008289)	-1.44
c50080	metal ion binding (GO:0046872)	-1.53
c55635	nucleic acid binding (GO:0003676)	-1.98
c58899	ATP binding (GO:0005524)	-2.76
c54701	double-stranded DNA binding (GO:0003690)	-2.77
c46084	binding (GO:0005488); protein binding (GO:0005515); unfolded protein binding (GO:0051082)	-2.98

† Inoculated refers to the library constructed by transcripts of *M. carassicauda* 12 h after conidial inoculation. Healthy refers to the library constructed from control treatment.

‡ FC means fold change of differentially expressed genes (DEGs) between the two libraries.

Table S5 The potential aphid proteins binding to CytCo by pull-down assay and LC-MS/MS

Unused	Coverage%	Peptides (95%)	Accession no.	Unigene ID	Nr Annotation
37.86	55.8	20	gi|1028717492	c55126	calcium-transporting ATPase sarcoplasmic/endoplasmic reticulum type isoform X2
21.79	66	17	gi|193596761	c58899	heat shock protein 68-like
21.74	61.3	12	gi|641653561	c59139	filamin-A isoform X1
19.52	76.2	11	gi|328703570	c55491	hexokinase type 2 isoform X3
16.71	61.9	9	gi|328709829	c57677	heterogeneous nuclear ribonucleoprotein 87F isoform X1
16.17	51.5	8	gi|641662999	c55861	coatomer subunit gamma-2 isoform X2
12.35	51.9	8	gi|328719143	c56749	actin-interacting protein 1 isoform X2
12.94	52.2	7	gi|1028703692	c55766	AMP deaminase 2 isoform X2
12.28	75.5	7	gi|240849631	c3867	ATP synthase subunit b, mitochondrial-like
9.94	55.9	6	gi|641659374	c54396	uncharacterized protein LOC100164133 isoform X1
10.1	63.8	6	gi|641658287	c56402	rab6-like isoform X1
10.44	37.5	5	gi|641662508	c52037	brain tumor protein
9.72	59.9	5	gi|641664799	c57640	synaptotagmin 1 isoform X1
7.8	54.8	5	gi|328700027	c57307	ATP-dependent 6-phosphofructokinase isoform X2
7.61	43.9	5	gi|328722529	c53806	uncharacterized protein C05D11.1
5.77	61.3	5	gi|254281298	c46865	small nuclear ribonucleoprotein E
10.55	51.7	4	gi|193573533	c58432	pre-mRNA-processing-splicing factor 8
8.64	71.1	4	gi|193606029	c52152	ras-related protein Rab-5C
6.98	44.1	4	gi|1028716082	c56786	glucose dehydrogenase
6.94	43.1	4	gi|328714905	c51674	dolichyl-diphosphooligosaccharide--protein glycosyltransferase 48 kDa subunit
6.78	75.4	4	gi|1685561546	c54691	ATPase family AAA domain-containing protein 3
6.28	51.4	4	gi|641666661	c54528	aspartate aminotransferase, cytoplasmic
5.52	54.6	4	gi|193657231	c57476	vesicle-fusing ATPase 1
1.97	49.5	4	gi|328718138	c57280	protein argonaute-2 isoform X2
6.8	59.3	3	gi|193587197	c50002	isocitrate dehydrogenase
6.82	53.6	3	gi|328715038	c56028	putative U5 small nuclear ribonucleoprotein 200 kDa helicase
6.79	81.1	3	gi|641653622	c55643	transcriptional activator protein Pur-beta-B isoform X2
6.25	74.8	3	gi|244790059	c49651	proteasome beta 2 subunit
6.15	56.2	3	gi|328698278	c53344	adenylosuccinate synthetase
6.13	52.2	3	gi|1685549878	c59125	fatty acyl-CoA reductase 1
6.1	60.6	3	gi|328699290	c52380	ATP-dependent RNA helicase vasa

6.11	43.7	3	gi|193676397	c58629	ATP-binding cassette sub-family F member 1
5.67	61.4	3	gi|328709119	c58703	lon protease homolog, mitochondrial
5.29	52.1	3	gi|641676995	c52852	dihydroorotate dehydrogenase (quinone), mitochondrial
4.64	52.2	3	gi|193650309	c57502	maternal protein exuperantia
4.65	35.5	3	gi|328717688	c54933	importin-4
4.64	67	3	gi|641656760	c58139	TAR DNA-binding protein 43
7.49	50.8	3	gi|328704956	c55057	aldo-keto reductase family 1 member B1
4.01	34.3	3	gi|193707023	c52282	ubiquitin-like modifier-activating enzyme 5
3.34	46.5	3	gi|641660278	c54609	serine/threonine-protein phosphatase 5
3.36	56.1	3	gi|240848631	c58797	glutathione S-transferase omega-1-like
2.03	62.8	3	gi|77415640	c50292	hypothetical protein
4.72	51.7	2	gi|641678204	c53264	tRNA (cytosine(34)-C(5))-methyltransferase
4.71	48.3	2	gi|641675516	c54607	SWI/SNF complex subunit SMARCC2 isoform X1
4.37	52.2	2	gi|328716117	c56903	ubiquitin carboxyl-terminal hydrolase 7 isoform X1
4.35	61.2	2	gi|193582327	c49523	uncharacterized protein LOC100166424
4.31	49.1	2	gi|193667016	c54332	replication protein A 70 kDa DNA-binding subunit
4.23	51	2	gi|641678166	c55131	probable hydroxyacid-oxoacid transhydrogenase, mitochondrial
4.1	41.8	2	gi|1685536842	c56310	LOW QUALITY PROTEIN: leukotriene A-4 hydrolase
4.1	58.3	2	gi|641676251	c57411	GTP-binding protein SAR1b
4.1	68.4	2	gi|193662169	c57149	U1 small nuclear ribonucleoprotein 70 kDa
4.1	33.9	2	gi|328723643	c53223	innexin inx7
4.03	63.4	2	gi|193584719	c58334	pseudouridylate synthase 7 homolog
4.02	65.5	2	gi|193636468	c58710	alpha-tocopherol transfer protein
4.01	53.6	2	gi|193652361	c57387	fumarylacetoacetate hydrolase domain-containing protein 2
4.01	9.9	2	gi|1028716222	c59449	transmembrane 9 superfamily member 2
4.12	55.3	2	gi|641658618	c53174	isoleucine--tRNA ligase, cytoplasmic
4.02	30.1	2	gi|328702264	c58847	scavenger receptor class B member 1 isoform X2
4.02	51.9	2	gi|1028717369	c55287	E3 UFM1-protein ligase 1 homolog
3.8	70.5	2	gi|242247429	c53425	SEC14 cytosolic factor-like
3.76	38.5	2	gi|240849422	c50205	NADH dehydrogenase

3.71	46.2	2	gi|328720151	c57885	protein Mo25
3.77	63.2	2	gi|641656969	c59744	ruvB-like helicase 1
3.62	59.3	2	gi|193671562	c51495	putative riboflavin kinase
3.46	49.8	2	gi|328717652	c56709	E3 SUMO-protein ligase RanBP2 isoform X1
3.44	46.3	2	gi|193582381	c57832	cell division cycle 5-like protein
3.31	61.1	2	gi|193629582	c58710	alpha-tocopherol transfer protein
3.2	44.2	2	gi|240849591	c58984	RNA-binding protein 8A-like
2.75	72.6	2	gi|240849091	c47099	MPA13 allergen-like
2.91	13.7	2	gi|193678939	c53648	organic cation transporter protein isoform X1
2.72	70.6	2	gi|193632108	c52343	UDP-N-acetylhexosamine pyrophosphorylase
2.74	38.2	2	gi|193603540	c58875	protein yellow
2.52	38.9	2	gi|328714616	c58982	UDP-glucuronosyltransferase 2B7
2.56	48.2	2	gi|641666422	c57405	peroxidase isoform X1
2.64	61.8	2	gi|641650241	c54663	succinate--CoA ligase
2.42	40.3	2	gi|1685534457	c59628	fatty acid synthase isoform X2
1.98	49.4	2	gi|193718449	c52643	phytoene desaturase
1.84	46.1	2	gi|193580180	c57529	sodium/potassium-transporting ATPase subunit beta-2
1.8	38.9	2	gi|328720239	c59036	regulator of microtubule dynamics protein 1 isoform X1
1.65	30.3	2	gi|641672251	c54081	tumor suppressor candidate 3

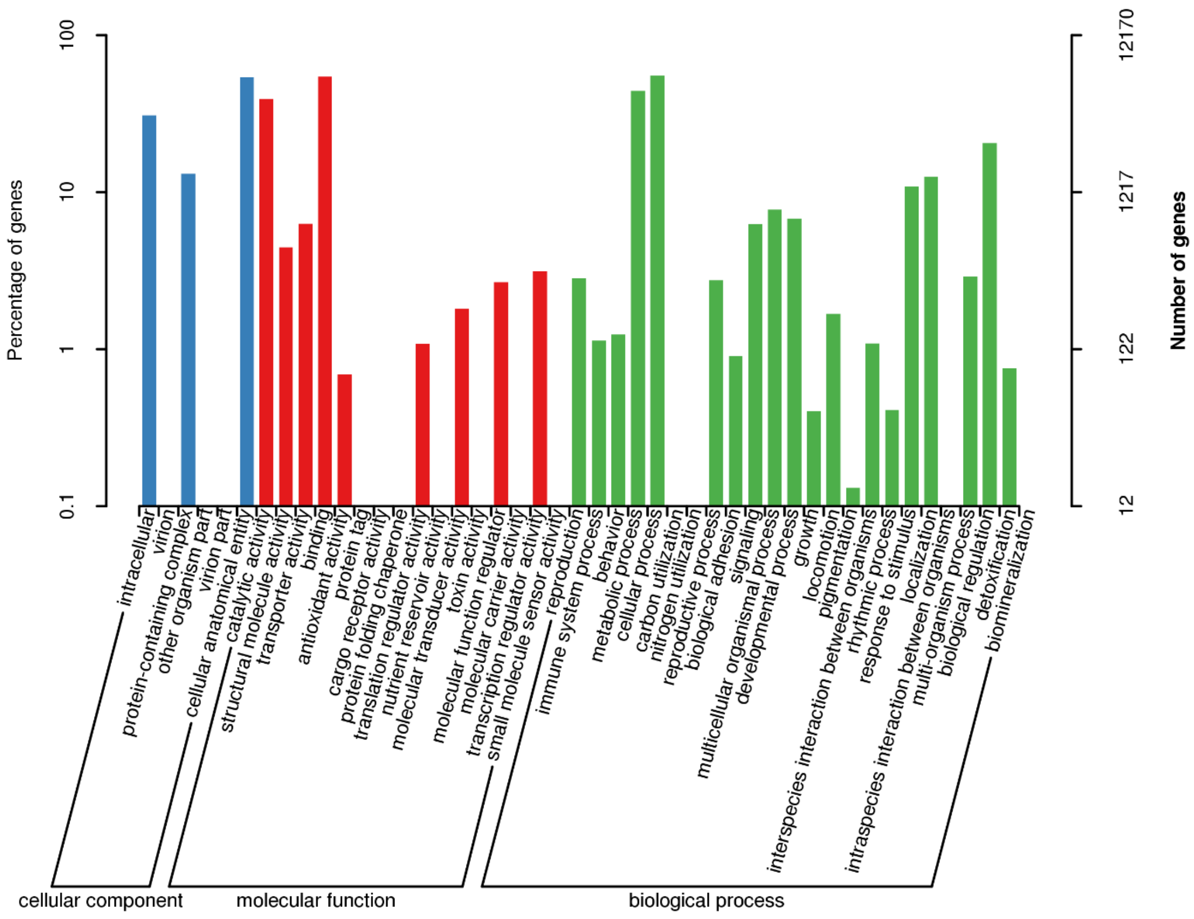

Figure S1: GO classification of DEG.

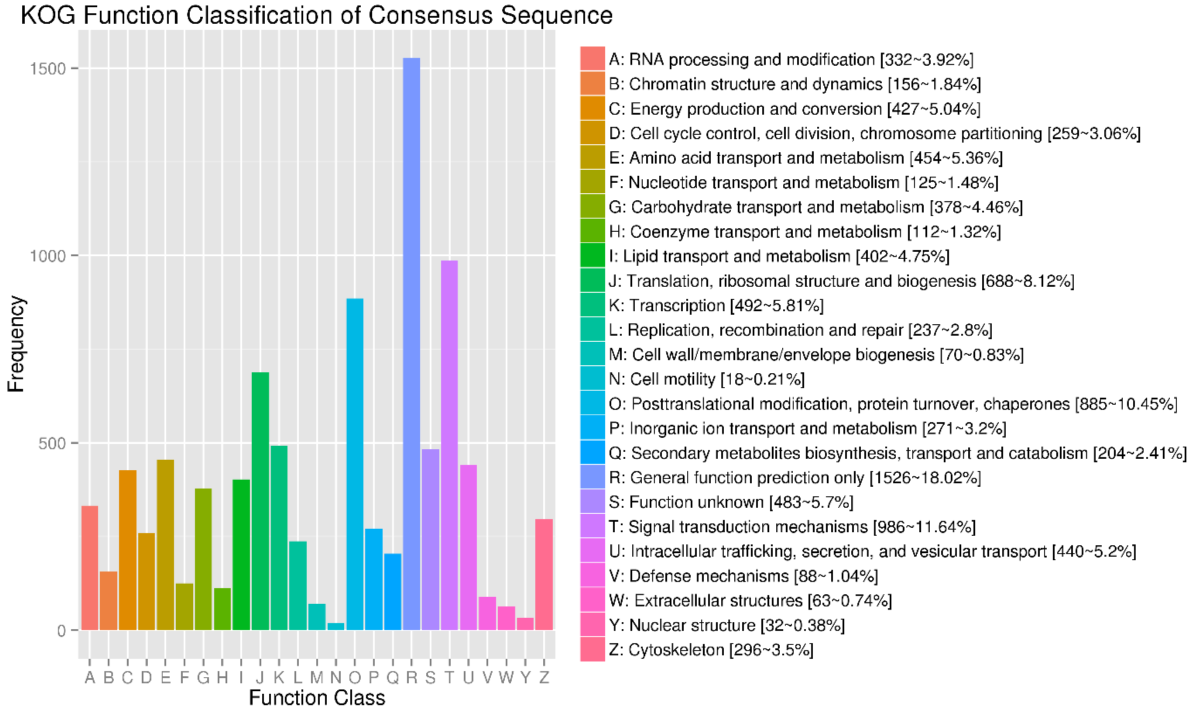

Figure S2: KOG Function Classification of Consensus Sequence.

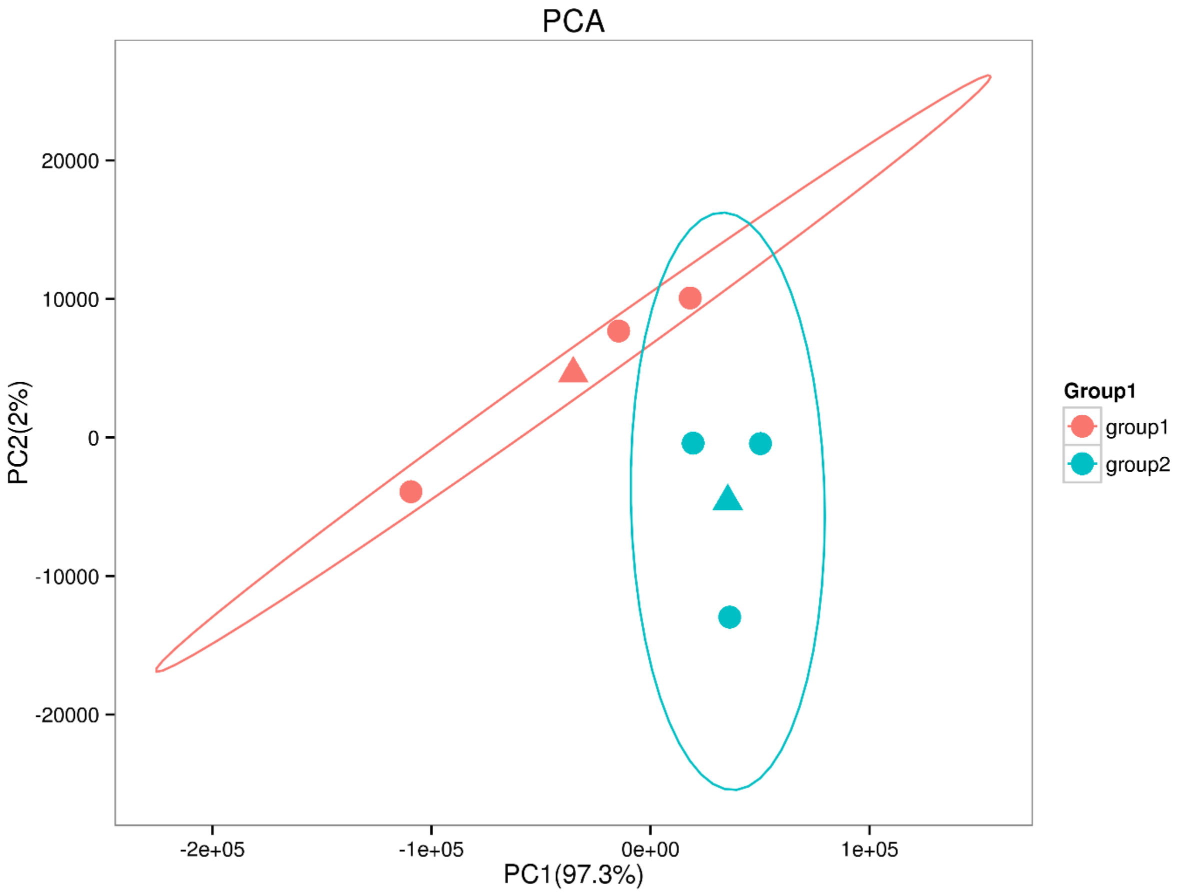

Figure. S3. PCA analysis of two group of samples. Group1: healthy aphids; group2: inoculated aphids.

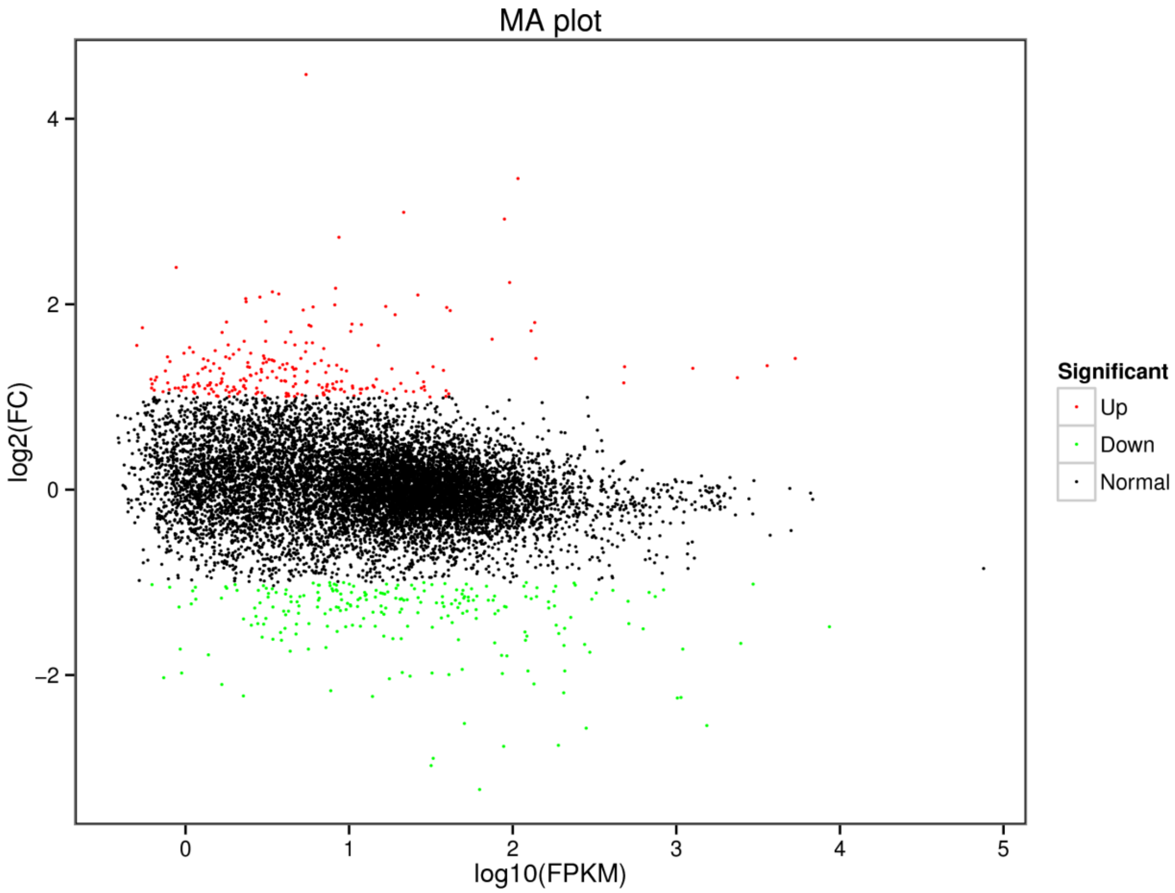

Figure. S4 Expression levels of the differentially expressed genes among the six samples of *Megoura crassicauda*. The scatter plot shows the relationship between log the fragments per kilobase per million fragments (log_2FPKM) and the log fold change (log_2FC) for each differentially expressed gene (FC≥2), which are based on the sequencing of the RNA that was extracted from the inoculated aphids when compared to the healthy aphids. Each symbol represents a single gene, the black circles indicate the genes without differential expression, while the colored symbols indicate genes that are differentially expressed at a false discovery rate of ≤0.05

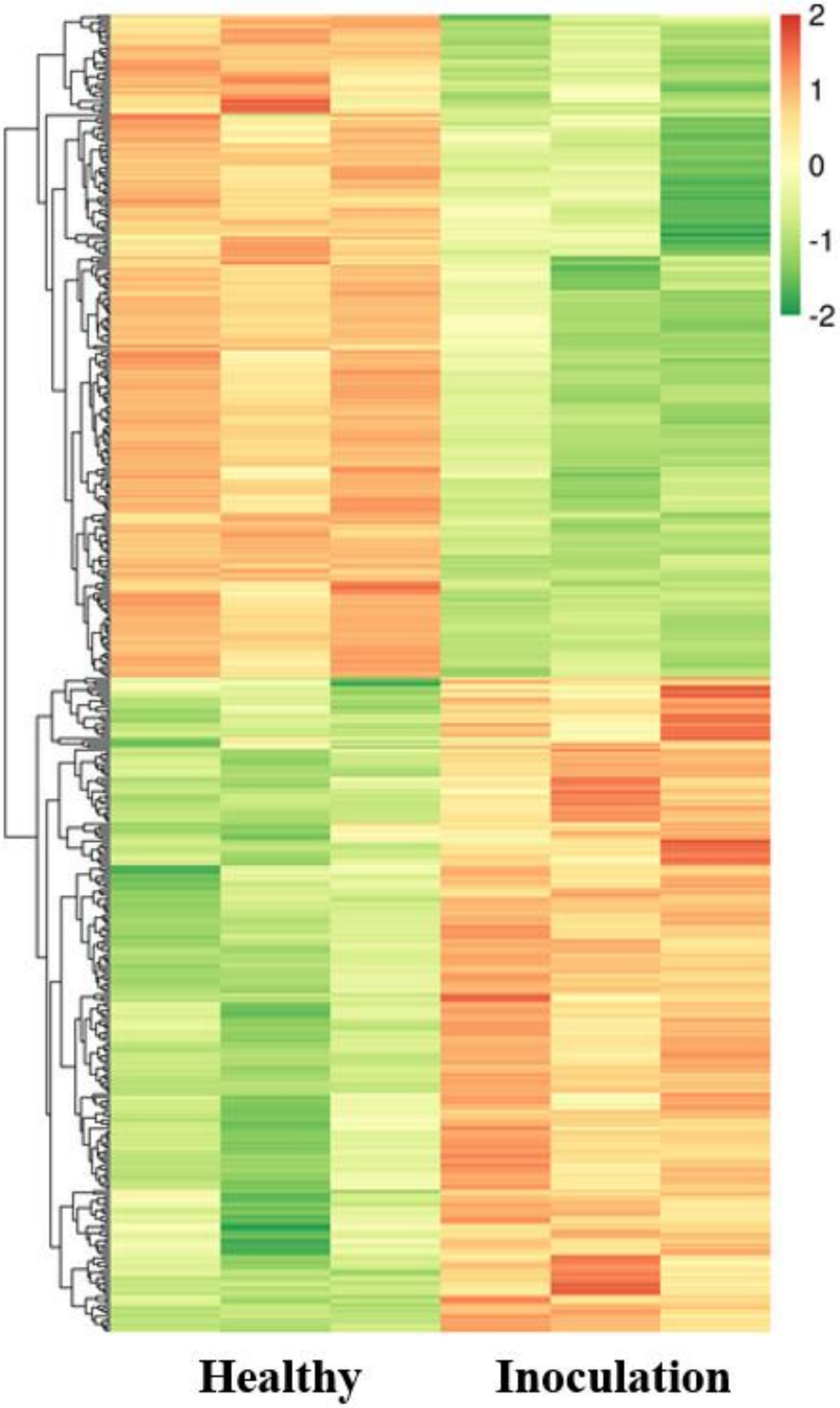

Figure. S5 Heat map of the transcription patterns of the differentially expressed genes among the six samples of *Megoura crassicauda*. The patterns were obtained from hierarchical clustering and the normalization of the $\log_{10}$ of the fragments per kilobase per million (FPKM), which were used to determine the gene expression levels across different samples.

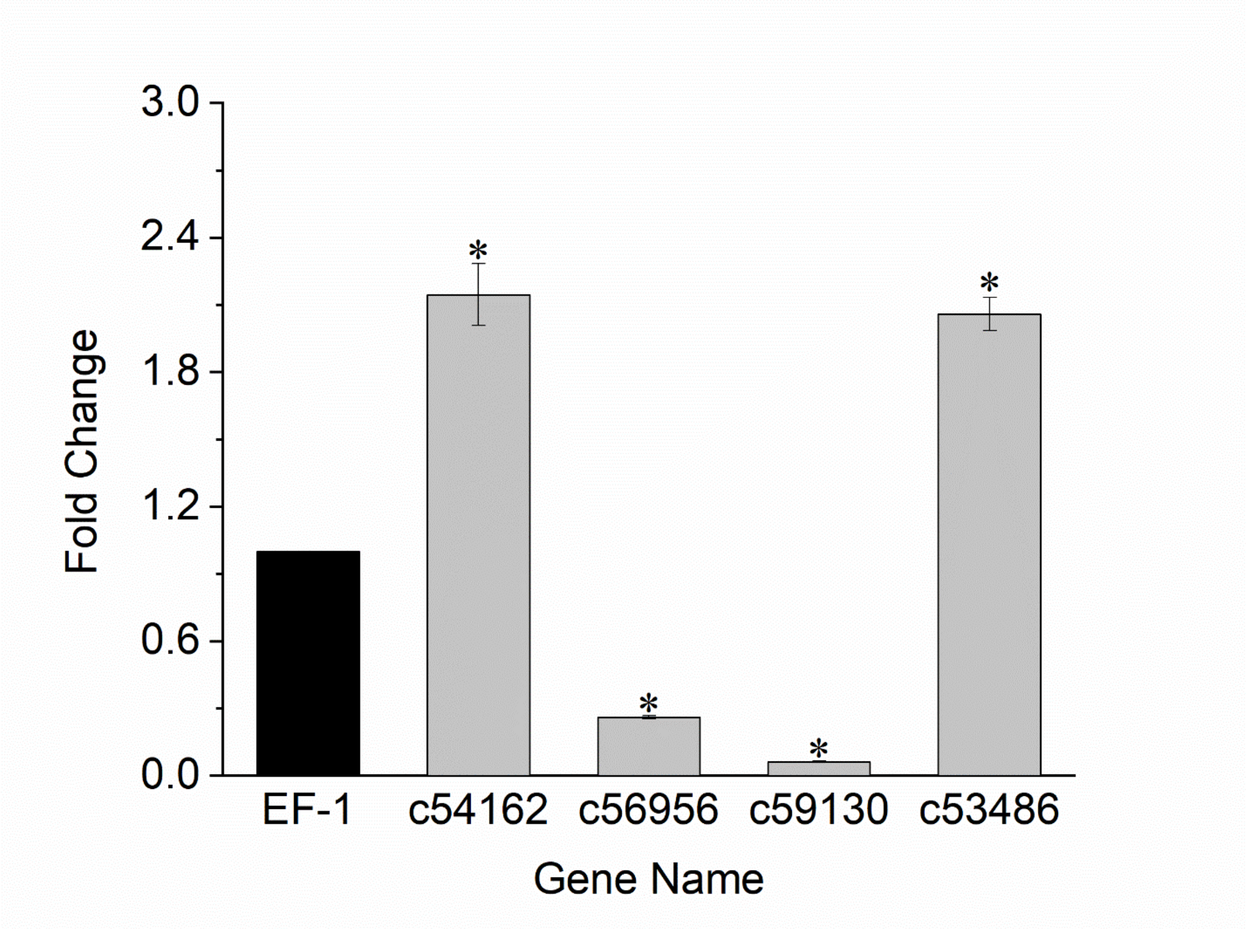

Figure. S6 RT-qPCR result of *Megoura crassicauda* DEGs' relative quantification, EF-1 is the reference gene, specifying the expression of EF-1 as 1, which is used to show the up-regulation and down-regulation of DEGs.

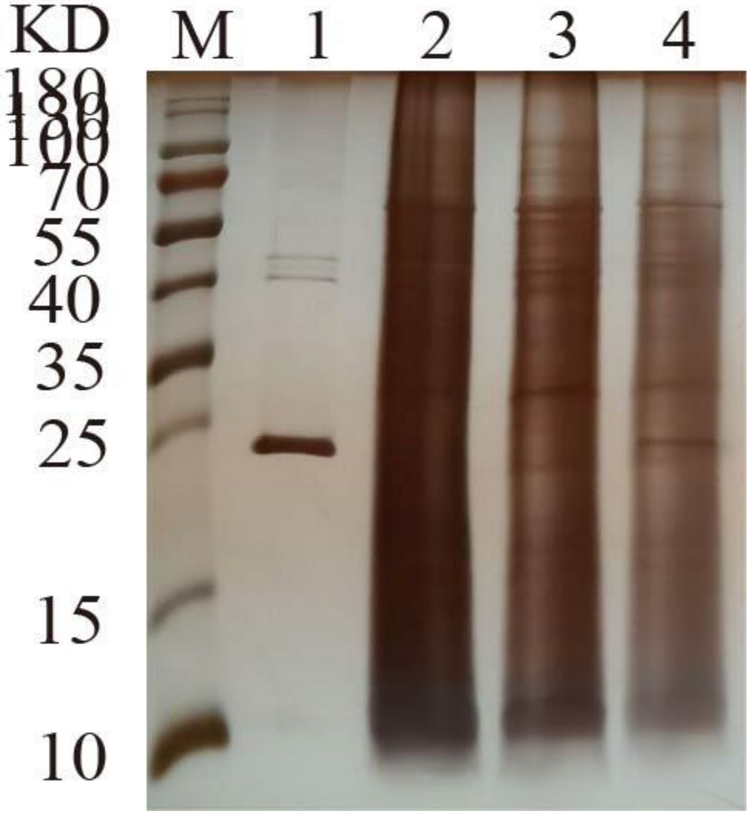

Figure S7. SDS-PAGE of aphid proteins and CytCo-binding interactors. Lane M protein standard; lane 1 CytCo protein; Lane 2 aphid total proteins; Lane3 His tag interact with aphid proteins; lane 4 CytCo interact with aphid proteins.

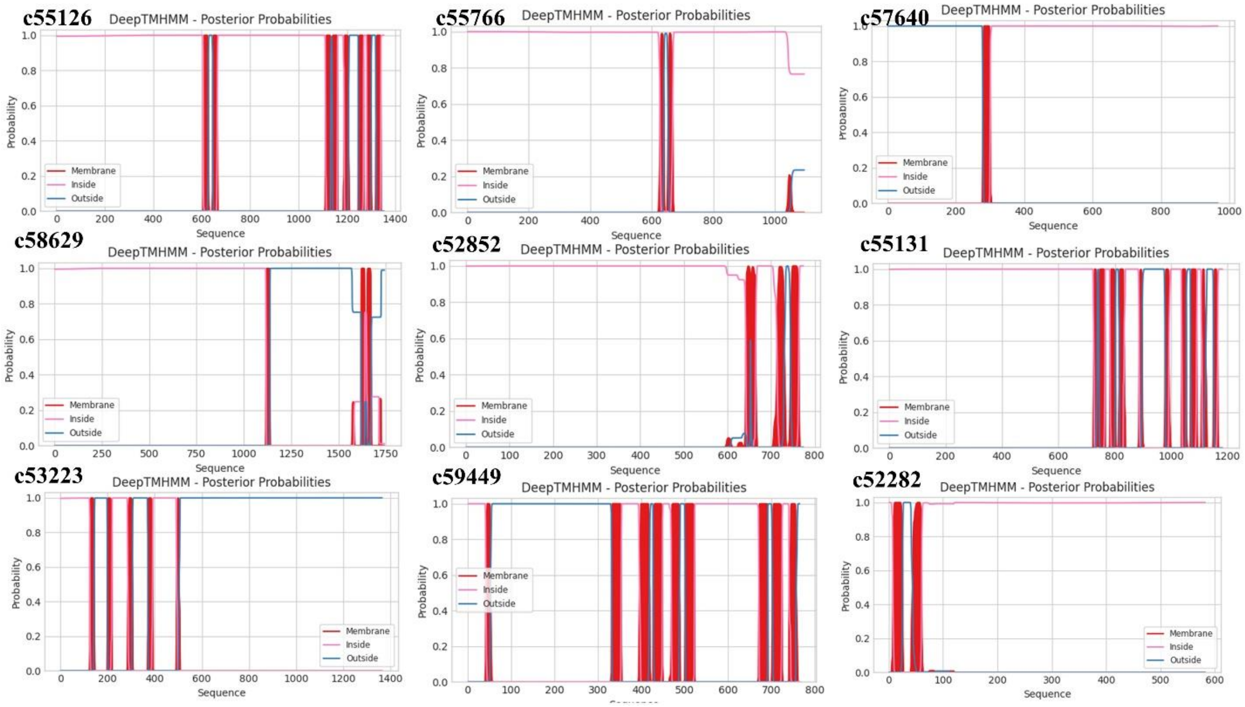

Figure S8. The transmembrane topology prediction of the potential CytCo-binding aphid proteins by DeepTMHMM (DTU/DeepTMHMM – BioLib).
